# The Influence of Intraoperative Technology on Neurosurgery Training

**DOI:** 10.7759/cureus.5769

**Published:** 2019-09-26

**Authors:** Alison Ho, Yasir R Khan, Eric Whitney, Anthony JG Alastra, Javed Siddiqi

**Affiliations:** 1 Neurosurgery, Desert Regional Medical Center, Palm Springs, USA

**Keywords:** intraoperative, neurosurgery, resident training, technology

## Abstract

Background

Intraoperative technology (IOT) is an expanding field designed to produce better patient outcomes and decrease iatrogenic injury. Neurosurgical residents often encounter these machines in the operating room. Therefore, our primary objective was to assess the influence of IOT on neurosurgery residents’ surgical skills and training.

Methods

An electronic survey was created and sent to the neurosurgical residency programs in the state of California. The data were collected and analyzed.

Results

A majority of residents agreed that IOT helps in learning new concepts important for patient safety. 38% agreed that IOT helps to improve the motivation level of residents. 35% agreed that IOT makes the resident more productive. 31% felt that IOT helped them refine their surgical skills. 54% did not find IOT too stressful. 34% said that IOT helps in learning new concepts important for patient safety. 50% agree that IOT is a valuable tool in training. 42% affirmed that IOT provides good learning experience for clinical skills and knowledge.

Conclusion

Surgical training and IOT have evolved substantially over the last decade and resulted in increased intraoperative accuracy. Residents agreed that evolving technology improves surgical skills. Further studies elucidating patient outcomes are warranted.

## Introduction

Humans interact with the environment using our five senses. With the rapid advancement in computer technology and engineering over the past fifty years, our ability to interact with the environment around us has evolved. Robotics allows us to move in spaces our fingers could not access and now with sub-millimetric precision. Navigation using radiographic images allows us to see within the human body and localize pathology before incisions are even made. In essence, our technology has become our sixth sense, guiding and improving upon our actions whether from weather forecasting using doppler radar, global positioning system (GPS) for finding the closest restaurant, or voice technology on our smartphones. Nowhere is this more evident than in the practice of medicine, especially the surgical sciences. There can be no doubt the current generation of practicing surgeons and surgical trainees have benefited from technological advancements [1]. Today computer-integrated robotic surgery provides additional information that was less available to surgeons through human senses alone. Additionally, new technology provides intelligent assistance to improve visualization and performance such as an overlay of a reconstructed computed tomography (CT) scan of a tumor on the operating site [2]. Neurosurgery has been a pioneer in adopting new technology. The PUMA 200 (Westinghouse Electric, Pittsburgh, PA) was the first robot that was used on humans in 1985 for needle placement in a CT-guided brain biopsy [3-4]. Neurosurgical training has continued to grow and develop with technology thus becoming an integral part of improving patient outcomes [5]. Several assistive techniques including fluorescence-guided resection, neuro-navigation, intraoperative magnetic resonance imaging (MRI)/CT stereotaxis, and neurophysiological monitoring have provided the neurosurgeon with better surgical results [6-7]. New technologies have become an essential tool in aiding a surgeon’s clinical judgment allowing them to perform many kinds of surgery with greater ease, accuracy, and efficacy [8]. The added confidence and improved visualization of anatomy and pathology have improved the quality and flow of surgery with fewer complications such as infections and major bleeding. Smaller incisions allow for a faster recovery with less pain and disfigurement [9].

The impact of intraoperative navigation guided technologies on neurosurgery residency training has yet to be quantified. Advanced technology in medical education has enhanced the education of core competencies among trainees resulting in educational aids that have the potential for optimizing the training process [10-11]. Casiano demonstrated that computer-assisted surgery has been improving the efficacy of endoscopic sinus surgery with fewer complications [12]. According to Martin et al., residents found that advanced surgical technology was integral or essential in 70% and was helpful in 20% [13]. In this study, we conducted a survey on the effects of technology with respect to neurosurgery resident training.

## Materials and methods

This study was exempted by the local Institutional Review Board and given an exemption letter. The survey and questionnaire were created using Survey Monkey (https://www.surveymonkey.com/r/3PTJS6B). This survey was sent to all neurosurgical residency programs in California from March to April 2018. The survey included questions which were designed to elicit the level of resident’s experience with intraoperative technology (IOT) and their perceived impact on training (Table 1).

**Table 1:** Questions asked in the survey Q: Question; PGY: Postgraduate Year

	Questions
Q1	Age
Q2	PGY
Q3	Intraoperative technology helps to improve the motivation level of residents.
Q4	Intraoperative technology makes residents more productive,
Q5	Intraoperative technology helps residents refine their surgical skills.
Q6	Intraoperative technology is too stressful.
Q7	Intraoperative technology helps residents in learning new concepts important for patient safety.
Q8	Intraoperative technology is a valuable tool in residents training.
Q9	Intraoperative technology provides good learning experience for clinical skills and knowledge.
Q10	Which intraoperative technology is available at your institute?

Q3-Q9 were answered using the 5-point Likert Scale, with choices of “strongly agree,” “agree,” “neutral,” “disagree,” and “strongly disagree.” Survey data were tabulated and reported as the percentage of respondents per specific option. Q10 was to assess the IOT present at the participants' institutions.

## Results

The beginning of the survey collected demographic data of the 26 respondents (Table 2).

**Table 2:** Participants demographics

Demographics	Number
Gender (Male:Female)	19:7
Post-Graduate Year	5:5:3:6:3:3:1

**Figure 1:**
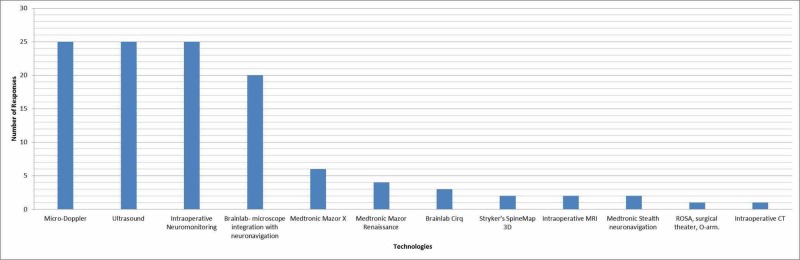
Intraoperative technologies available at the institutes of the respondents

The last question in the survey gathered information about the availability of various intraoperative technologies. As seen in Figure 1, 96.15% of the respondents had micro-doppler, ultrasound, and intraoperative neuromonitoring. 76.92% had Brainlab microscope integration with neuronavigation (Brainlab USA, Redwood City, CA), 23.08% had Medtronic Mazor X (Medtronic and Mazor Robotics, Memphis, TN), 15.38% had Medtronic Mazor Renaissance (Mazor Robotics, Caesarea, Israel), 11.54% had Brainlab Cirq (Brainlab, Munich, Germany), 7.69% had Stryker SpineMap 3D (Stryker, Kalamazoo, MI), intraoperative MRI, and Medtronic Stealth neuronavigation (Medtronic Inc., Minnesota, MI), and 3.84% had intraoperative CT and Robotic Stereotactic Assistance (ROSA, MedTech Surgical Inc., Newark, NJ), surgical theater, and O-arm.

**Figure 2:**
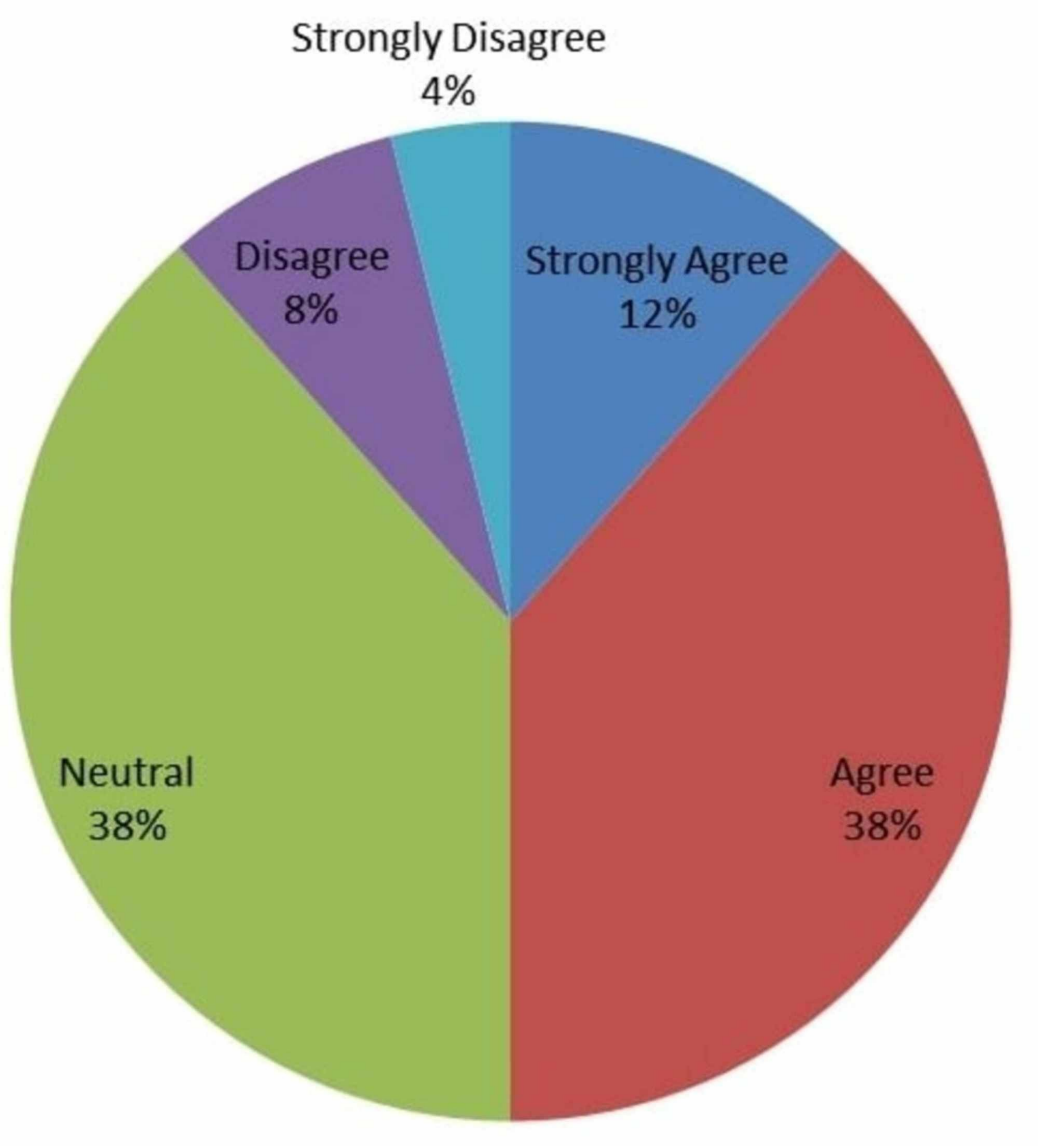
Responses to whether intraoperative technology improved motivation level of residents

**Figure 3:**
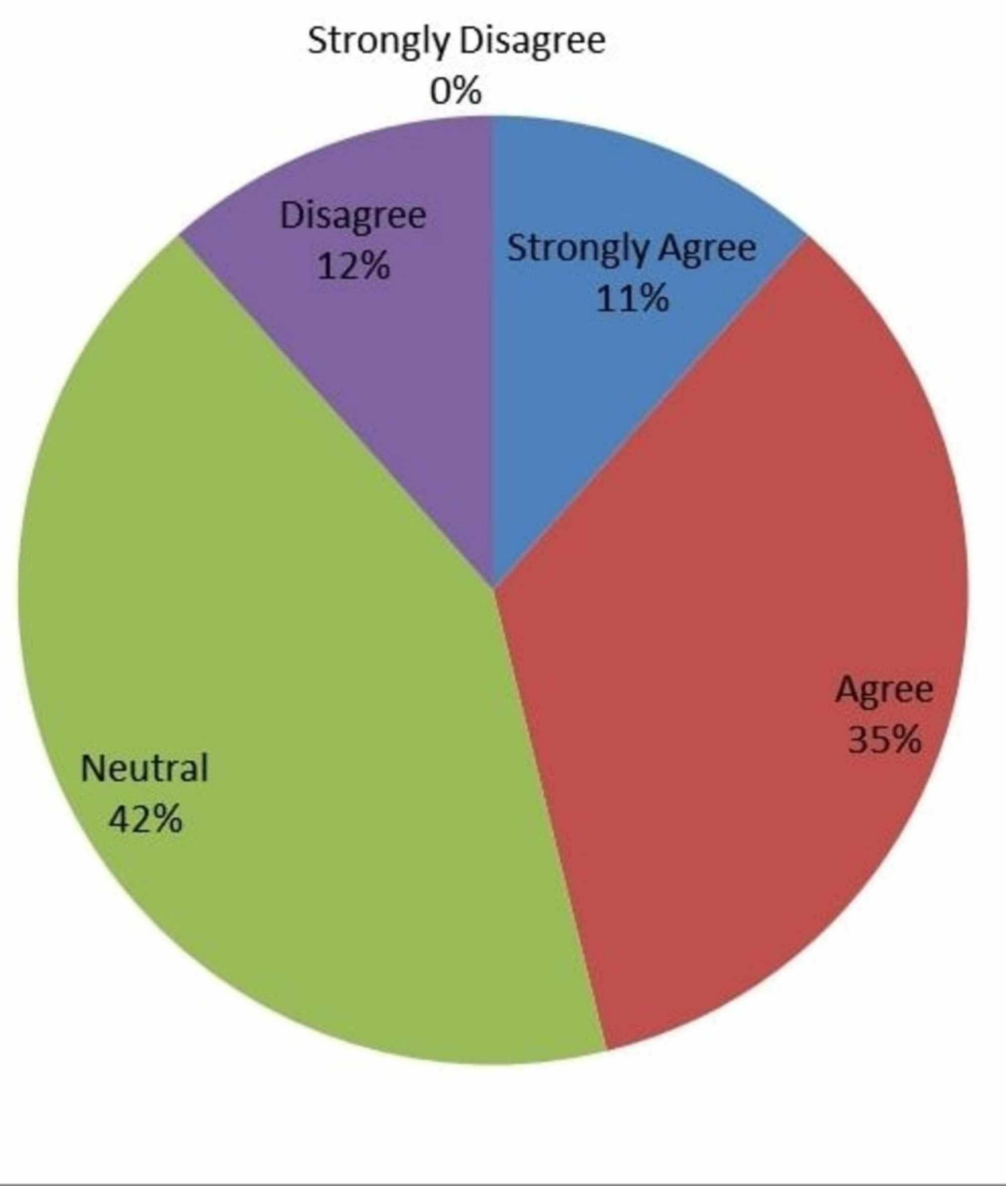
Responses to whether intraoperative technology improved productivity level of residents

**Figure 4:**
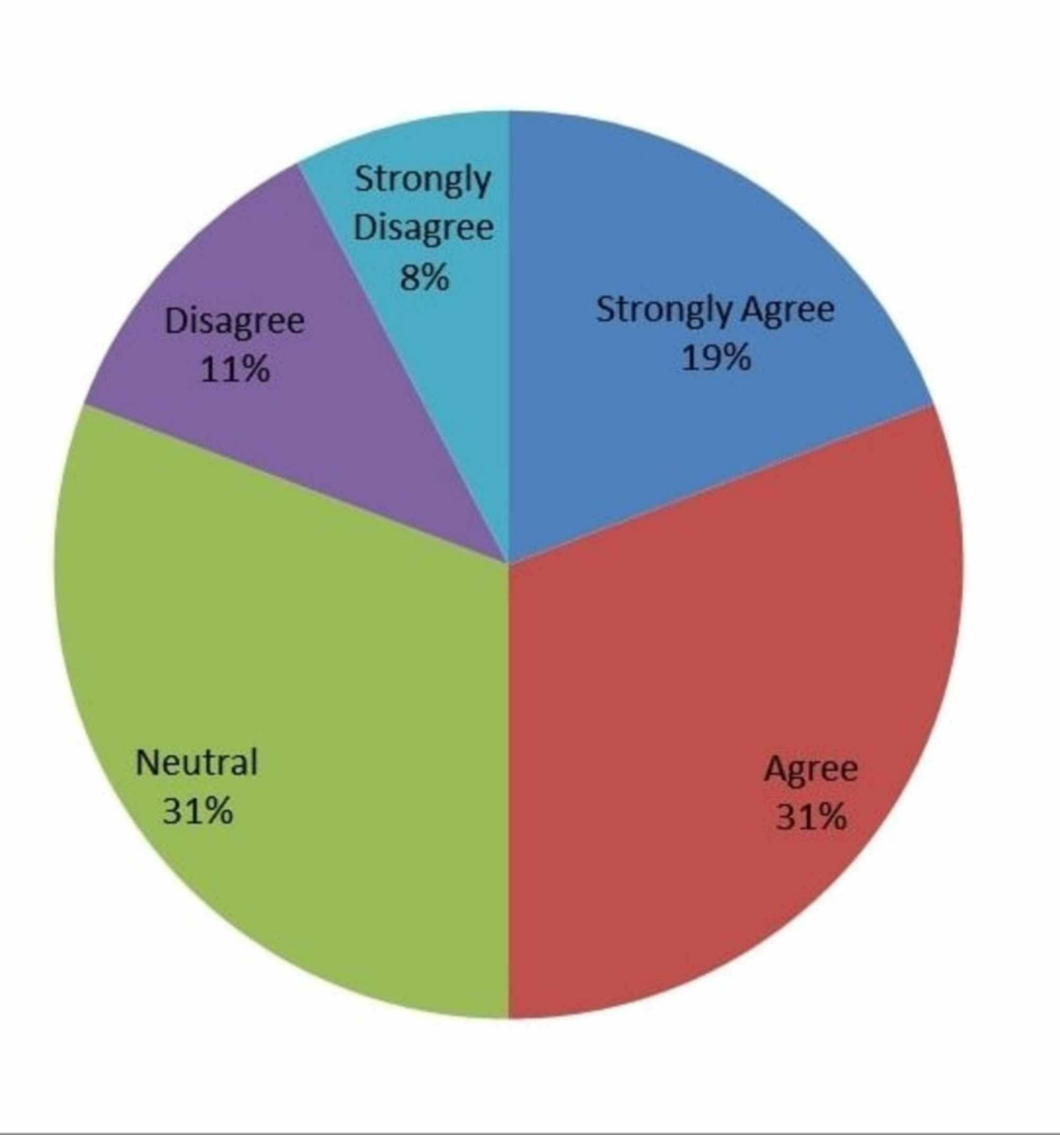
Responses to whether intraoperative technology improved surgical skill level of residents

**Figure 5:**
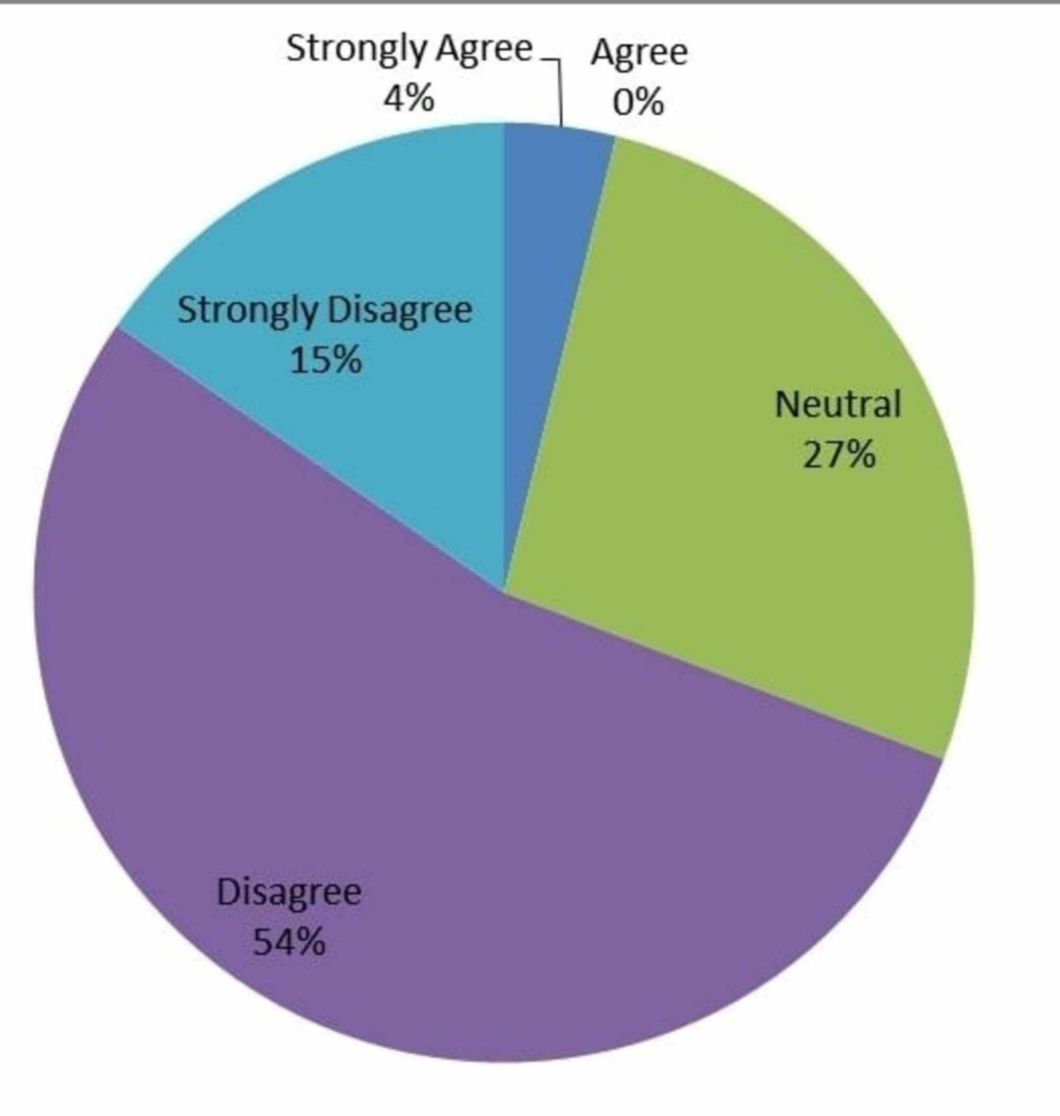
Responses to whether intraoperative technology was too stressful to use

**Figure 6:**
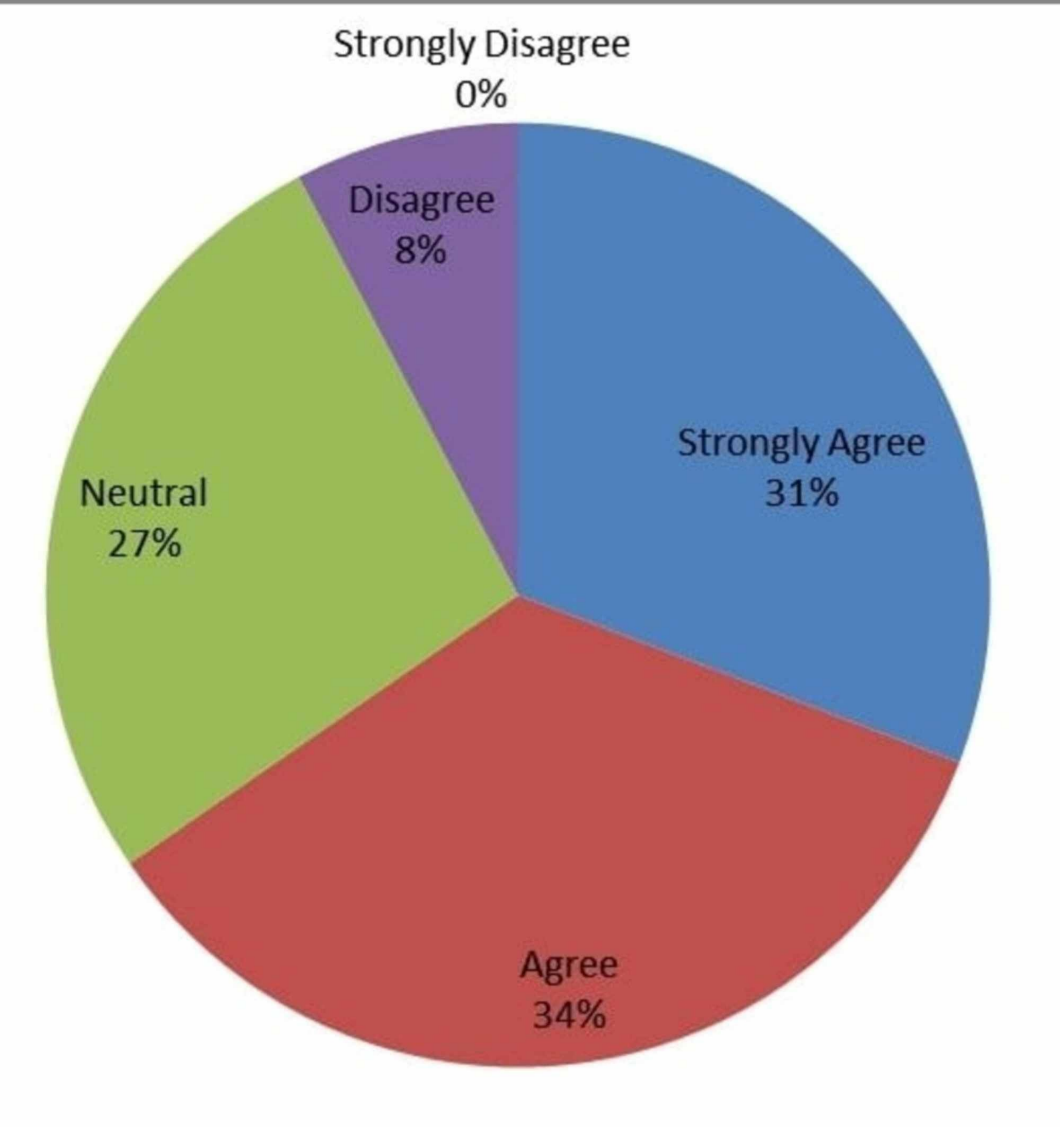
Responses to whether intraoperative technology helped learn new concepts with regards to patient safety

**Figure 7:**
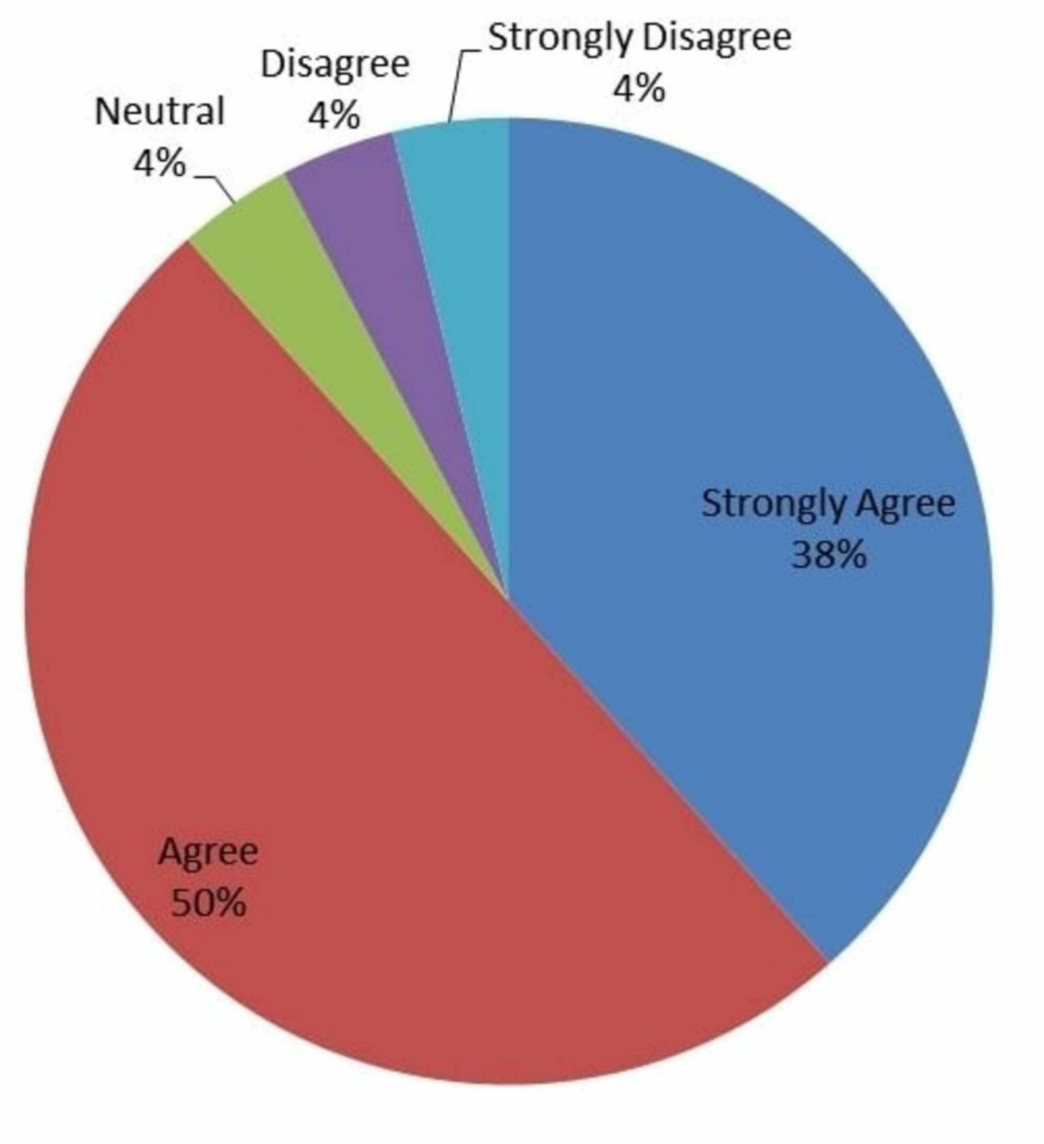
Responses to whether intraoperative technology was valuable to resident training

**Figure 8:**
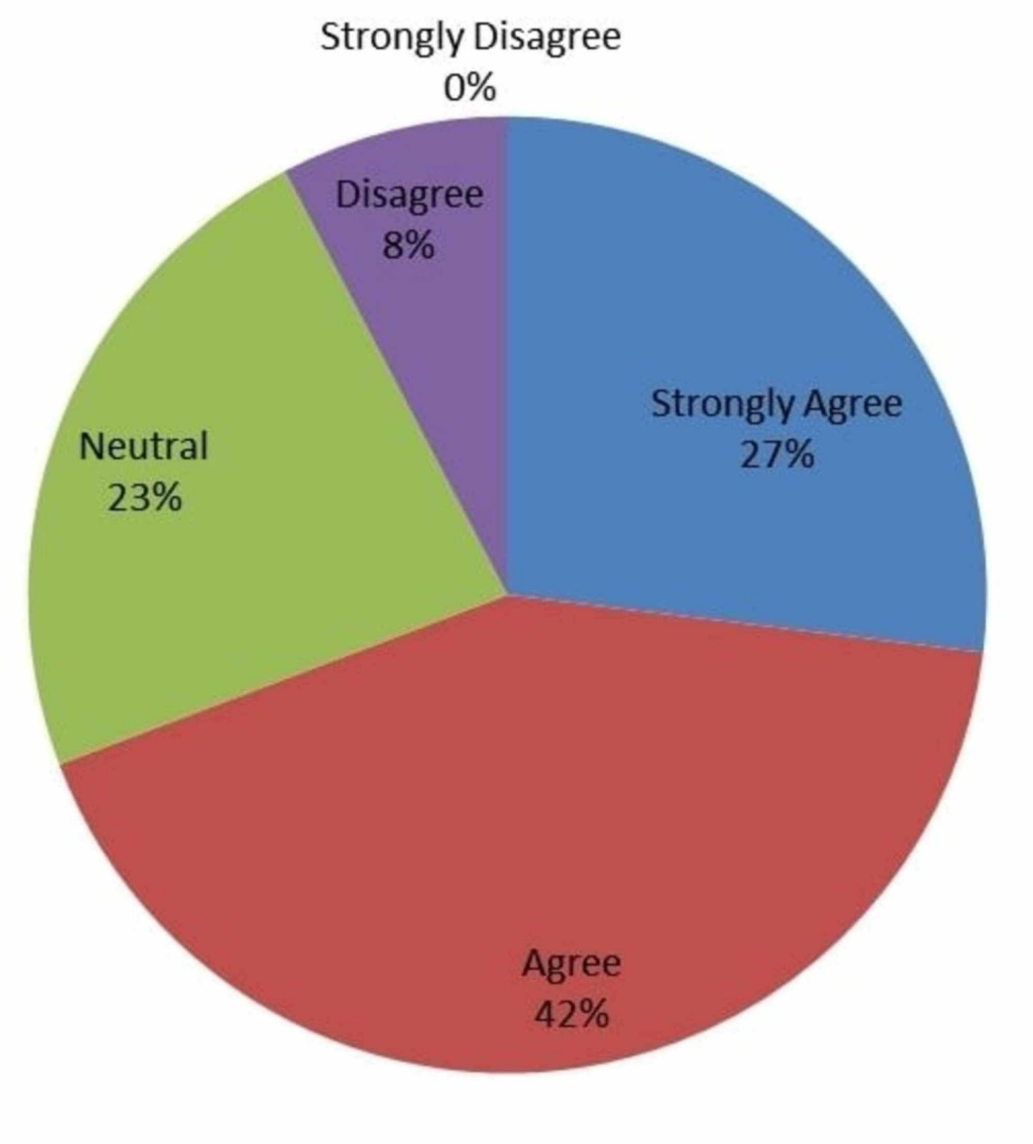
Responses to whether intraoperative technology provides a good learning experience with regards to clinical skills and knowledge

The rest of the questions elicited opinions regarding a variety of aspects of the use and availability of intraoperative technologies. Figures 2-8 outline the results of each question as a percentage of the respondents who chose each answer option. As seen in these figures, a majority of respondents agreed that IOT helps residents in learning new concepts important for patient safety, is a valuable tool in resident training, and provides a good learning experience for clinical skills and knowledge. Only 50% of respondents agreed that IOT increased motivation levels of residents and surgical skill level. Most respondents felt neutral towards a perceived increase in productivity. The majority of residents disagreed that IOT was too stressful to use. As seen in Figure 2, 12% (n=3) strongly agreed, 38% (n=10) agreed, 38% (n=10) were neutral, 8% (n=2) disagreed, and 4% (n=1) strongly disagreed that IOT helps to improve the motivation level of residents.

Figure 3 displays the resident responses regarding whether IOT encourages productivity where 11% (n=3) strongly agreed, 35% (n=9) agreed, 42% (n=11) and 12% (n=3) disagreed.

When it came to whether residents felt that IOT helped them refine their surgical skills, 19% (n=5) strongly agreed, 31% (n=8) agreed, 31% (n=8) were neutral, 11% (n=3) disagreed, and 8% (n=2) strongly disagreed (Figure 4). No specific skill was targeted but rather a general broad use of the term was questioned.

A broad general question asked if they find IOT too stressful; Figure 5 shows that 4% (n=1) strongly agreed, 27% (n=7) were neutral, 54% (n=14) disagreed, and 15% (n=4) strongly disagreed. This question did not target the reason why some felt it was “stressful” nor did it address the outcomes of said “stress”.

As seen in Figure 6, 31% (n=8) strongly agree, 34% (n=9) agree, 27% (n=7) were neutral and 8% (n=2) disagreed that IOT helps residents in learning new concepts important for patient safety.

Figure 7 showed that 38% (n=10) strongly agree, 50% (n=13) agree, 4% (n=1) were neutral, 4% (n=1) disagree, and 4% (n=1) strongly disagreed that IOT is a valuable tool in residents training.

As seen in Figure 8, 27% (n=7) strongly agreed, 42% (n=11) agreed, 23% (n=6) were neutral, and 8% (n=2) disagreed that IOT provides good learning experience for clinical skills and knowledge.

## Discussion

Technology is believed to reinforce surgical skills without compromising patient safety [14]. A majority of residents found intraoperative technologies available at their medical center to be a valuable tool in their training and provided an improvement in clinical skills, knowledge, and patient safety. This could be attributed to increased ease with performing surgery, leading to an increased focus on more technical skills that are also vital to surgical training without necessarily leading to a decline in the less technical skills. Overall, a majority of residents felt that IOT provided a good learning experience in implementing technology to address current pathology. This is likely due to the use of an additional modality to further visualize pathology and provide another frame of reference for evaluation. These technologies, therefore, help one “see” what one is operating on better than one’s own eyes alone.

Only half of the residents agreed or strongly agreed that better technology improved motivation or surgical skill level. Neurosurgery residents could be considered motivated individuals regardless of available technology, amenities, or conveniences. One may be lead to believe that intraoperative tools may become ‘crutches’, reducing the necessity to perform surgery based on anatomical landmarks and prior imaging for the patient and residents could find themselves in situations without available technology and feel less comfortable performing the same surgeries. Fewer than half of the residents agreed that IOT increased productivity; only a small minority disagreed with that statement. These advanced surgical tools may be seen as a foundational aspect of each case, meaning productivity would be an inherent feature of all available equipment, including intraoperative imaging technology. Rather than being seen as increasing productivity, these tools are simply necessary to effectively and safely complete the surgery.

The survey did reveal a small group of residents who found the incorporation of IOT to neurosurgical training to be stressful. This could be due to a lack of quality training and potential sources of error involved with the operational complexity of the equipment available. It has been shown that comprehensive systematic stepwise training curriculum beginning with online video didactics, then hands-on tutorial simulation, culminating in real-time performance are necessary for residents to be proficient at robotic-assisted intraoperative procedures as well as decreased trainee frustration [15-18]. Therefore, in order to have a quality benefit, a comprehensive formal training curriculum should be established [19]. As described by Schulz et al., an error can occur during registration, skin marking, or anatomical landmarks may be inaccurate due to scalp movement, geometrical distortion in the images, and movement of the patient with respect to the system during surgery. Once surgery has begun and the cranial vault has been opened, brain shift (movement of the brain relative to the cranium between the time of navigational scanning and the time of surgery) can render navigation tools off course by several millimeters or more [20]. Medical centers generally have a variety of options when it comes to imaging modalities, so learning the intricacies and nuances of each one adds to the vast amount of knowledge required by the neurosurgery resident.

Micro-doppler, ultrasound, and intraoperative neuromonitoring were found to be available as a part of many neurosurgery-training programs. Along with the results from the remainder of the survey, it is clear that these tools are specifically useful to residents. However, a broad stroke recommendation as to which specific product that should be used cannot be made with the available information from the survey.

Limitations

The number of responses received from the survey was small. The survey was only sent to neurosurgical residencies in California, limiting the survey to a representation of 12 programs in one state. These results may still give an indication as to resident opinion outside of this region but would need to be refined to accommodate regional differences (if any) in technologies available at specific medical institutions.

## Conclusions

Surgical training and IOT have evolved substantially over the last decade and have resulted in increased intraoperative accuracy. As a part of neurosurgery residency, these tools have become a vital part of residency training. From this study, it is clear that a program’s investment in these technologies is not only valuable to the attending surgeons' surgical prowess, but also to the residents' quality of training. Future studies will be needed to investigate which products provide the greatest cost benefit for institutions and elucidate information on improved patient safety and reduction in iatrogenic injury.
